# Ethyl 2-[(3-chloro­phen­yl)hydrazono]-3-oxobutanoate

**DOI:** 10.1107/S160053680901784X

**Published:** 2009-05-20

**Authors:** Hoong-Kun Fun, Mahesh Padaki, Sowmya, Arun M. Isloor, Suchada Chantrapromma

**Affiliations:** a X-ray Crystallography Unit, School of Physics, Universiti Sains Malaysia, 11800 USM, Penang, Malaysia; b Department of Chemistry, National Institute of Technology-Karnataka, Surathkal, Mangalore 575 025, India; c Crystal Materials Research Unit, Department of Chemistry, Faculty of Science, Prince of Songkla University, Hat-Yai, Songkhla 90112, Thailand

## Abstract

The mol­ecule of the title oxobutanoate derivative, C_12_H_13_ClN_2_O_3_, adopts a keto–hydrazo tautomeric form and is roughly planar, the angle between the benzene ring and the mean plane through the hydrazone and aliphatic chain being 1.49 (6)°. This planarity is further aided by the formation of an intra­molecular N—H⋯O hydrogen bond which generates an *S*(6) ring motif. The aromatic ring and aliphatic chain have a *trans* configuration with respect to the N—N bond. In the crystal packing, centrosymmetric *R*_2_^2^(16) dimers are formed through pairs of weak C—H⋯O(3-oxo) inter­actions. These dimers are linked together through weak C—H⋯O(carboxyl­ate C=O) inter­actions into ribbons along the *b-*axis direction. These ribbons are stacked along the *a*-axis direction. The crystal also exhibits Cl⋯Cl [3.4988 (6) Å] and C⋯O [3.167 (2)–3.335 (2) Å] short contacts.

## Related literature

For bond-length data, see: Allen *et al.* (1987 ▶). For hydrogen-bond motifs, see: Bernstein *et al.* (1995 ▶). For background to the bioactivity and applications of oxobutanoate derivatives, see: Alpaslan *et al.* (2005*a* ▶,*b* ▶); Stancho *et al.* (2008 ▶). For related structures, see: Alpaslan *et al.* (2005*a* ▶,*b* ▶); Fun *et al.* (2009 ▶). For the stability of the temperature controller used in the data collection, see: Cosier & Glazer, (1986 ▶).
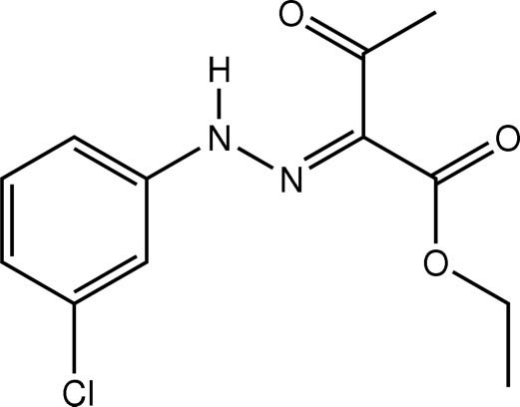


## Experimental

### 

#### Crystal data

C_12_H_13_ClN_2_O_3_
*M* *_r_* = 268.69
Triclinic, 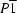

*a* = 4.0826 (2) Å
*b* = 10.3196 (4) Å
*c* = 15.1469 (6) Å
α = 88.336 (3)°
β = 87.033 (3)°
γ = 83.734 (2)°
*V* = 633.31 (5) Å^3^
*Z* = 2
Mo *K*α radiation
μ = 0.30 mm^−1^
*T* = 120 K
0.39 × 0.11 × 0.06 mm

#### Data collection

Bruker APEXII CCD area-detector diffractometer
Absorption correction: multi-scan (*SADABS*; Bruker, 2005 ▶) *T*_min_ = 0.890, *T*_max_ = 0.981
11030 measured reflections
3678 independent reflections
2732 reflections with *I* > 2σ(*I*)
*R*_int_ = 0.036

#### Refinement

*R*[*F* ^2^ > 2σ(*F* ^2^)] = 0.049
*wR*(*F* ^2^) = 0.139
*S* = 1.05
3678 reflections
169 parameters
H atoms treated by a mixture of independent and constrained refinement
Δρ_max_ = 0.75 e Å^−3^
Δρ_min_ = −0.27 e Å^−3^

### 

Data collection: *APEX2* (Bruker, 2005 ▶); cell refinement: *SAINT* (Bruker, 2005 ▶); data reduction: *SAINT*; program(s) used to solve structure: *SHELXTL* (Sheldrick, 2008 ▶); program(s) used to refine structure: *SHELXTL*; molecular graphics: *SHELXTL*; software used to prepare material for publication: *SHELXTL* and *PLATON* (Spek, 2009 ▶).

## Supplementary Material

Crystal structure: contains datablocks global, I. DOI: 10.1107/S160053680901784X/fb2150sup1.cif

Structure factors: contains datablocks I. DOI: 10.1107/S160053680901784X/fb2150Isup2.hkl

Additional supplementary materials:  crystallographic information; 3D view; checkCIF report

## Figures and Tables

**Table 1:** Hydrogen-bond geometry (Å, °)

*D*—H⋯*A*	*D*—H	H⋯*A*	*D*⋯*A*	*D*—H⋯*A*
N1—H1*N*1⋯O3	0.91 (3)	1.87 (3)	2.564 (2)	132 (3)
C3—H3*A*⋯O1^i^	0.93	2.54	3.211 (2)	129
C5—H5*A*⋯O3^ii^	0.93	2.52	3.433 (2)	166

Symmetry codes: (i) 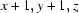; (ii) 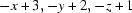.

**Fig. 1.**
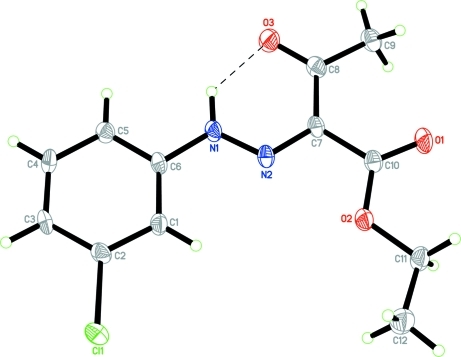
A view of the title molecule, showing 50% probability displacement ellipsoids and the atom-numbering scheme. The N—H···O hydrogen bond is depicted as a dashed line.

**Fig. 2.**
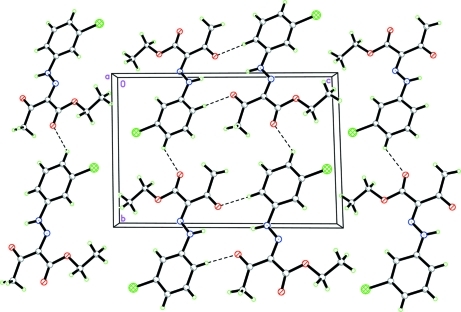
The packing diagram of the title compound, viewed along the a axis, showing the molecular ribbons. Hydrogen bonds are drawn as dashed lines.

## supplementary crystallographic information

### Comment

In recent years, the chemistry of hydrazones have been the subject of intense study mostly due to their biological significance. Some oxobutanoate derviatives exhibit cytotoxicity (Stancho *et al.*, 2008). We previously reported the crystal structure of the ethyl 2-[(4-chlorophenyl)hydrazono]-3-oxobutanoate (I) (Fun *et al.*, 2009). As part of our on going research on the synthesis and biological activity of oxobutanoates, we report here the synthesis and crystal structure of the title compound, ethyl 2-[(3-chlorophenyl)hydrazono]-3-oxobutanoate (II).

The molecule of the title oxobutanoate derivative (II), C_12_H_13_ClN_2_O_3_, adopts a keto-hydrazo tautomeric form and is roughly planar as indicated by the interplanar angle between the benzene ring and the mean plane through the hydrazone and aliphatic chain (N1–N2/O1–O3/C7–C12) being 1.49 (6)°. The aromatic ring and aliphatic chain have a *trans* configuration with respect to the N—N bond as evidenced by the torsion angle C6–N1–N2–C7 being 179.76 (15)°. The orientations of 3-oxobutanoate and ethyl group are determined by the torsion angles C10–C7–C8–C9 = 3.3 (3)° and C10–O2–C11–C12 = 168.38 (10)° [the corresponding angles are 2.81 (15)° and 170.6 (9)° in (I) (Fun *et al.*, 2009)]. The intramolecular N1—H1···O1 hydrogen bond generates an S(6) ring motif (Bernstein *et al.*, 1995) (Table 1). The bond distances in (II) have normal values (Allen *et al.*, 1987) and are comparable to those in closely related structures (Alpaslan *et al.*, 2005*a*, *b*; Fun *et al.*, 2009).

In the crystal packing (Fig. 2), the molecules are present as centrosymmetric *R*_2_^2^(16) dimers being joined by weak, centrosymmetrically related C5—H5A···O3 interactions involving the 3-oxo group (Table 1). These dimers are linked together through weak C3—H3A···O1  (carboxylate C═O) interactions (Table 1) into ribbons along the *b* direction.  These ribbons are stacked along the *a* direction. The crystal also shows Cl···Cl [3.4988 (6) Å; symmetry code: 2 - *x*, 3 - *y*, -*z*] and C···O [3.167 (2)–3.335 (2) Å; symmetry code: -1 + *x*, *y*, *z*] short contacts.

### Experimental

The title compound was prepared by disolving 3-chloroaniline (1.27 g, 10 mmol) in dilute hydrochloric acid (11.0 ml), obtained by mixing 4.5 ml of 12 M HCl and 6.5 ml water. The solution was cooled to 273 K in ice bath. To this, a cold solution of sodium nitrite (1.6 g, 23.1 mmol in 5.0 ml water) was added. The temperature of reaction mixture was not allowed to rise above 323 K. The diazonium salt solution so formed was poured through a filter into a cooled solution of ethylacetoacetate (1.7 ml) and sodium acetate (3.5 g) in ethanol (50 ml). The resulting yellow solid was filtered, washed with ice cold water, dried in air and recrystallized from methanol. Yield was found to be 1.70 g (70 %), *M.p.* 360 K.

### Refinement

The hydrazone H atom was located in a difference map and refined isotropically. The remaining H atoms were placed in calculated positions with d(C—H) = 0.93 Å for aromatic, 0.97 for CH_2_ and 0.96 Å for CH_3_ atoms. The *U*_iso_ values were constrained to be 1.5*U*_eq_ of the carrier atom for methyl H atoms and 1.2*U*_eq_ for the remaining H atoms. A rotating group model was used for the methyl groups. The highest residual electron density peak is located at 0.86 Å from Cl1 and the deepest hole is located at 1.19 Å from C2. The difference electron density map also indicated possible tautomerism with the docking site (N2). However, the ^1^H NMR experiments did not confirm this tautomerism. Moreover it would be difficult to model a resonance structure that would be in agreement with the presumed tautomerism.

### Crystal data

**Table:**

C_12_H_13_ClN_2_O_3_	*Z* = 2
*M**_r_* = 268.69	*F*(000) = 280
Triclinic, *P*1	*D*_x_ = 1.409 Mg m^−^^3^
Hall symbol: -P 1	Melting point: 360 K
*a* = 4.0826 (2) Å	Mo *K*α radiation, λ = 0.71073 Å
*b* = 10.3196 (4) Å	Cell parameters from 3678 reflections
*c* = 15.1469 (6) Å	θ = 1.4–30.0°
α = 88.336 (3)°	µ = 0.30 mm^−^^1^
β = 87.033 (3)°	*T* = 120 K
γ = 83.734 (2)°	Needle, yellow
*V* = 633.31 (5) Å^3^	0.39 × 0.11 × 0.06 mm

### Data collection

**Table:**

Bruker APEXII CCD area-detector diffractometer	3678 independent reflections
Radiation source: sealed tube	2732 reflections with *I* > 2σ(*I*)
graphite	*R*_int_ = 0.036
φ and ω scans	θ_max_ = 30.0°, θ_min_ = 1.4°
Absorption correction: multi-scan (*SADABS*; Bruker, 2005)	*h* = −5→5
*T*_min_ = 0.890, *T*_max_ = 0.981	*k* = −14→14
11030 measured reflections	*l* = −18→21

### Refinement

**Table:**

Refinement on *F*^2^	Primary atom site location: structure-invariant direct methods
Least-squares matrix: full	Secondary atom site location: difference Fourier map
*R*[*F*^2^ > 2σ(*F*^2^)] = 0.049	Hydrogen site location: inferred from neighbouring sites
*wR*(*F*^2^) = 0.139	H atoms treated by a mixture of independent and constrained refinement
*S* = 1.05	*w* = 1/[σ^2^(*F*_o_^2^) + (0.0778*P*)^2^ + 0.1165*P*] where *P* = (*F*_o_^2^ + 2*F*_c_^2^)/3
3678 reflections	(Δ/σ)_max_ = 0.001
169 parameters	Δρ_max_ = 0.75 e Å^−^^3^
0 restraints	Δρ_min_ = −0.27 e Å^−^^3^

### Special details

**Table:**

Experimental. The crystal was placed in the cold stream of an Oxford Cryosystems Cobra open-flow nitrogen cryostat (Cosier & Glazer, 1986) operating at 120.0 (1) K.
Geometry. All e.s.d.'s (except the e.s.d. in the dihedral angle between two l.s. planes) are estimated using the full covariance matrix. The cell e.s.d.'s are taken into account individually in the estimation of e.s.d.'s in distances, angles and torsion angles; correlations between e.s.d.'s in cell parameters are only used when they are defined by crystal symmetry. An approximate (isotropic) treatment of cell e.s.d.'s is used for estimating e.s.d.'s involving l.s. planes.
Refinement. Refinement of *F*^2^ against ALL reflections. The weighted *R*-factor *wR* and goodness of fit *S* are based on *F*^2^, conventional *R*-factors *R* are based on *F*, with *F* set to zero for negative *F*^2^. The threshold expression of *F*^2^ > σ(*F*^2^) is used only for calculating *R*-factors(gt) *etc*. and is not relevant to the choice of reflections for refinement. *R*-factors based on *F*^2^ are statistically about twice as large as those based on *F*, and *R*- factors based on ALL data will be even larger.

### Fractional atomic coordinates and isotropic or equivalent isotropic displacement parameters (Å^2^)

**Table:**

	*x*	*y*	*z*	*U*_iso_*/*U*_eq_	
Cl1	1.05405 (13)	1.38226 (4)	0.08494 (3)	0.02972 (15)	
O1	0.6525 (4)	0.66454 (13)	0.29235 (9)	0.0307 (3)	
O2	0.6912 (3)	0.82739 (12)	0.19234 (8)	0.0233 (3)	
O3	1.2031 (4)	0.86080 (13)	0.46009 (9)	0.0300 (3)	
N1	1.1619 (4)	1.03691 (14)	0.33708 (10)	0.0199 (3)	
N2	0.9988 (3)	0.95997 (13)	0.29359 (10)	0.0189 (3)	
C1	1.1244 (4)	1.20062 (16)	0.21705 (11)	0.0207 (3)	
H1A	1.0155	1.1475	0.1826	0.025*	
C2	1.1889 (4)	1.32389 (17)	0.18746 (12)	0.0207 (3)	
C3	1.3528 (4)	1.40505 (16)	0.23737 (12)	0.0226 (4)	
H3A	1.3904	1.4880	0.2163	0.027*	
C4	1.4591 (4)	1.35961 (17)	0.31912 (12)	0.0229 (4)	
H4A	1.5732	1.4120	0.3528	0.027*	
C5	1.3972 (4)	1.23677 (16)	0.35144 (12)	0.0206 (3)	
H5A	1.4676	1.2069	0.4065	0.025*	
C6	1.2282 (4)	1.15919 (16)	0.30004 (11)	0.0185 (3)	
C7	0.9369 (4)	0.84466 (16)	0.32803 (11)	0.0190 (3)	
C8	1.0544 (4)	0.79195 (17)	0.41344 (12)	0.0222 (4)	
C9	1.0059 (5)	0.65572 (17)	0.44501 (13)	0.0260 (4)	
H9A	1.1162	0.6367	0.4991	0.039*	
H9B	1.0961	0.5950	0.4010	0.039*	
H9C	0.7745	0.6484	0.4551	0.039*	
C10	0.7486 (4)	0.76823 (16)	0.27083 (11)	0.0201 (3)	
C11	0.5170 (5)	0.75610 (17)	0.13154 (12)	0.0248 (4)	
H11A	0.3238	0.7247	0.1613	0.030*	
H11B	0.6596	0.6819	0.1089	0.030*	
C12	0.4174 (5)	0.8497 (2)	0.05750 (14)	0.0331 (4)	
H12A	0.3063	0.8057	0.0148	0.050*	
H12B	0.6103	0.8817	0.0297	0.050*	
H12C	0.2719	0.9214	0.0807	0.050*	
H1N1	1.211 (6)	1.016 (3)	0.3940 (17)	0.043 (7)*	

### Atomic displacement parameters (Å^2^)

**Table:**

	*U*^11^	*U*^22^	*U*^33^	*U*^12^	*U*^13^	*U*^23^
Cl1	0.0420 (3)	0.0212 (2)	0.0261 (2)	−0.00346 (18)	−0.00707 (18)	0.00680 (16)
O1	0.0434 (8)	0.0159 (6)	0.0353 (7)	−0.0126 (6)	−0.0079 (6)	0.0038 (5)
O2	0.0281 (6)	0.0170 (6)	0.0259 (6)	−0.0066 (5)	−0.0059 (5)	0.0027 (5)
O3	0.0436 (8)	0.0185 (6)	0.0290 (7)	−0.0051 (6)	−0.0111 (6)	0.0037 (5)
N1	0.0242 (7)	0.0112 (6)	0.0246 (8)	−0.0018 (5)	−0.0046 (6)	0.0020 (5)
N2	0.0187 (7)	0.0114 (6)	0.0264 (7)	−0.0006 (5)	−0.0016 (6)	−0.0006 (5)
C1	0.0244 (8)	0.0139 (8)	0.0245 (8)	−0.0038 (6)	−0.0033 (7)	−0.0013 (6)
C2	0.0242 (8)	0.0145 (8)	0.0225 (8)	0.0019 (6)	−0.0025 (7)	0.0036 (6)
C3	0.0271 (9)	0.0108 (7)	0.0303 (9)	−0.0052 (6)	−0.0001 (7)	0.0018 (6)
C4	0.0249 (9)	0.0138 (8)	0.0310 (9)	−0.0042 (6)	−0.0045 (7)	−0.0044 (7)
C5	0.0220 (8)	0.0158 (8)	0.0238 (8)	0.0000 (6)	−0.0039 (6)	0.0007 (6)
C6	0.0198 (8)	0.0105 (7)	0.0249 (8)	−0.0006 (6)	0.0001 (6)	0.0008 (6)
C7	0.0203 (8)	0.0114 (7)	0.0253 (8)	−0.0016 (6)	−0.0012 (6)	0.0014 (6)
C8	0.0250 (8)	0.0149 (8)	0.0257 (9)	0.0009 (6)	−0.0005 (7)	0.0019 (6)
C9	0.0306 (9)	0.0157 (8)	0.0310 (9)	−0.0004 (7)	−0.0026 (7)	0.0052 (7)
C10	0.0195 (8)	0.0153 (8)	0.0254 (8)	−0.0014 (6)	−0.0012 (6)	0.0005 (6)
C11	0.0276 (9)	0.0185 (8)	0.0292 (9)	−0.0049 (7)	−0.0045 (7)	−0.0031 (7)
C12	0.0368 (11)	0.0286 (10)	0.0349 (11)	−0.0045 (8)	−0.0116 (9)	0.0023 (8)

### Geometric parameters (Å, °)

**Table:**

Cl1—C2	1.7432 (18)	C4—H4A	0.9300
O1—C10	1.210 (2)	C5—C6	1.391 (2)
O2—C10	1.340 (2)	C5—H5A	0.9300
O2—C11	1.455 (2)	C7—C8	1.472 (2)
O3—C8	1.241 (2)	C7—C10	1.485 (2)
N1—N2	1.303 (2)	C8—C9	1.502 (2)
N1—C6	1.414 (2)	C9—H9A	0.9600
N1—H1N1	0.91 (3)	C9—H9B	0.9600
N2—C7	1.330 (2)	C9—H9C	0.9600
C1—C2	1.384 (2)	C11—C12	1.501 (3)
C1—C6	1.388 (2)	C11—H11A	0.9700
C1—H1A	0.9300	C11—H11B	0.9700
C2—C3	1.390 (2)	C12—H12A	0.9600
C3—C4	1.385 (2)	C12—H12B	0.9600
C3—H3A	0.9300	C12—H12C	0.9600
C4—C5	1.390 (2)		
			
C10—O2—C11	115.77 (13)	C8—C7—C10	121.60 (14)
N2—N1—C6	120.27 (15)	O3—C8—C7	119.29 (15)
N2—N1—H1N1	119.6 (16)	O3—C8—C9	118.90 (16)
C6—N1—H1N1	119.7 (16)	C7—C8—C9	121.80 (15)
N1—N2—C7	120.48 (15)	C8—C9—H9A	109.5
C2—C1—C6	117.59 (15)	C8—C9—H9B	109.5
C2—C1—H1A	121.2	H9A—C9—H9B	109.5
C6—C1—H1A	121.2	C8—C9—H9C	109.5
C1—C2—C3	122.41 (16)	H9A—C9—H9C	109.5
C1—C2—Cl1	119.40 (13)	H9B—C9—H9C	109.5
C3—C2—Cl1	118.17 (13)	O1—C10—O2	123.08 (16)
C4—C3—C2	118.50 (15)	O1—C10—C7	124.24 (16)
C4—C3—H3A	120.8	O2—C10—C7	112.67 (14)
C2—C3—H3A	120.8	O2—C11—C12	106.71 (14)
C3—C4—C5	120.83 (15)	O2—C11—H11A	110.4
C3—C4—H4A	119.6	C12—C11—H11A	110.4
C5—C4—H4A	119.6	O2—C11—H11B	110.4
C4—C5—C6	118.93 (16)	C12—C11—H11B	110.4
C4—C5—H5A	120.5	H11A—C11—H11B	108.6
C6—C5—H5A	120.5	C11—C12—H12A	109.5
C1—C6—C5	121.71 (15)	C11—C12—H12B	109.5
C1—C6—N1	121.53 (15)	H12A—C12—H12B	109.5
C5—C6—N1	116.75 (15)	C11—C12—H12C	109.5
N2—C7—C8	124.09 (15)	H12A—C12—H12C	109.5
N2—C7—C10	114.25 (15)	H12B—C12—H12C	109.5
			
C6—N1—N2—C7	179.76 (15)	N1—N2—C7—C8	−2.8 (3)
C6—C1—C2—C3	0.4 (3)	N1—N2—C7—C10	179.91 (14)
C6—C1—C2—Cl1	−178.31 (13)	N2—C7—C8—O3	4.8 (3)
C1—C2—C3—C4	0.9 (3)	C10—C7—C8—O3	−178.06 (16)
Cl1—C2—C3—C4	179.64 (13)	N2—C7—C8—C9	−173.83 (16)
C2—C3—C4—C5	−1.3 (3)	C10—C7—C8—C9	3.3 (3)
C3—C4—C5—C6	0.4 (3)	C11—O2—C10—O1	−2.8 (2)
C2—C1—C6—C5	−1.3 (3)	C11—O2—C10—C7	178.04 (14)
C2—C1—C6—N1	177.53 (16)	N2—C7—C10—O1	−175.72 (16)
C4—C5—C6—C1	0.9 (3)	C8—C7—C10—O1	6.9 (3)
C4—C5—C6—N1	−177.98 (16)	N2—C7—C10—O2	3.4 (2)
N2—N1—C6—C1	−0.3 (2)	C8—C7—C10—O2	−173.96 (15)
N2—N1—C6—C5	178.63 (15)	C10—O2—C11—C12	168.38 (16)

### Hydrogen-bond geometry (Å, °)

**Table:**

*D*—H···*A*	*D*—H	H···*A*	*D*···*A*	*D*—H···*A*
N1—H1N1···O3	0.91 (3)	1.87 (3)	2.564 (2)	132 (3)
C3—H3A···O1^i^	0.93	2.54	3.211 (2)	129
C5—H5A···O3^ii^	0.93	2.52	3.433 (2)	166

Symmetry codes:  (i) *x*+1, *y*+1, *z*; (ii) −*x*+3, −*y*+2, −*z*+1.
